# When lymph defies benign surgery: postoperative chylous fistula

**DOI:** 10.1093/jscr/rjaf1117

**Published:** 2026-01-22

**Authors:** Gustavo A León, Alvaro S Lemarie, Washington J Cisneros, Sofia A Velasco, Erika D Montenegro, Tatiana P Campoverde

**Affiliations:** Department of General Surgery, Hospital Padre Carollo “Un Canto a la Vida”, Av. Rumichaca S33-10, 170702 Quito, Ecuador; Department of General Surgery, Hospital Padre Carollo “Un Canto a la Vida”, Av. Rumichaca S33-10, 170702 Quito, Ecuador; Department of General Surgery, Hospital Padre Carollo “Un Canto a la Vida”, Av. Rumichaca S33-10, 170702 Quito, Ecuador; Department of General Surgery, Hospital Padre Carollo “Un Canto a la Vida”, PGY4 Pontificia Universidad Católica del Ecuador, Av. Rumichaca S33-10, 170702 Quito, Ecuador; Department of General Surgery, Hospital Padre Carollo “Un Canto a la Vida”, PGY4 Pontificia Universidad Católica del Ecuador, Av. Rumichaca S33-10, 170702 Quito, Ecuador; Department of General Surgery, Hospital Padre Carollo “Un Canto a la Vida”, PGY3 Pontificia Universidad Católica del Ecuador, Av. Rumichaca S33-10, 170702 Quito, Ecuador

**Keywords:** chylous fistula, abdominal surgery, benign pathology, complications, conservative management

## Abstract

Postoperative chylous fistula in abdominal surgery for benign pathologies is an extremely rare complication. This condition usually develops after extensive resections, chest or neck surgery, and is characterized by lymphatic leakage outside the abdominal cavity. In the following report, we describe two cases of patients undergoing non-oncologic abdominal surgery who developed a chylous fistula during the postoperative period and who responded adequately to conservative management.

## Introduction

Chylous fistula is a rare complication of abdominal surgery. The incidence of this condition varies between 0.3% and 6% depending on the procedure performed. It has been seen more frequently in procedures that include wide resections with lymph node dissection, such as right colectomies with lymphadenectomy, pancreatic surgery, abdominal surgeries in the context of trauma, aortic aneurysm, and thoracic surgery [1, 2].

Here we report two cases of chylous fistulas in abdominal surgery for benign pathology in elderly male patients in which their resolution was through conservative management.

## Case 1

A 68-year-old male with a history of hypothyroidism, gastroesophageal reflux disease (GERD) treated with omeprazole, esophageal candidiasis, and open prostatectomy. He presented with abdominal pain of 3 months' duration, predominantly in the right hypochondrium. Complementary tests showed no inflammatory response. Abdominal and pelvic tomography (CT) diagnosed a segment II liver abscess. He remained hospitalized on an antibiotic regimen, but symptoms worsened and were accompanied by vomiting and abdominal distension. A new CT scan was performed, which showed free perihepatic fluid, distension of intestinal loops, and a double halo sign.

An exploratory laparotomy was performed, revealing a 60 cm segment of small intestine 55 cm from the angle of Treitz with signs of ischemia without perforation. A resection and isoperistaltic side-to-side anastomosis were performed. Postoperative management included multimodal analgesia, antibiotic therapy with ceftriaxone and metronidazole for suspected liver abscess, and bowel rest for manipulation and type II bowel dysfunction, which was managed with ambulation and prokinetics.

**Figure 1:**
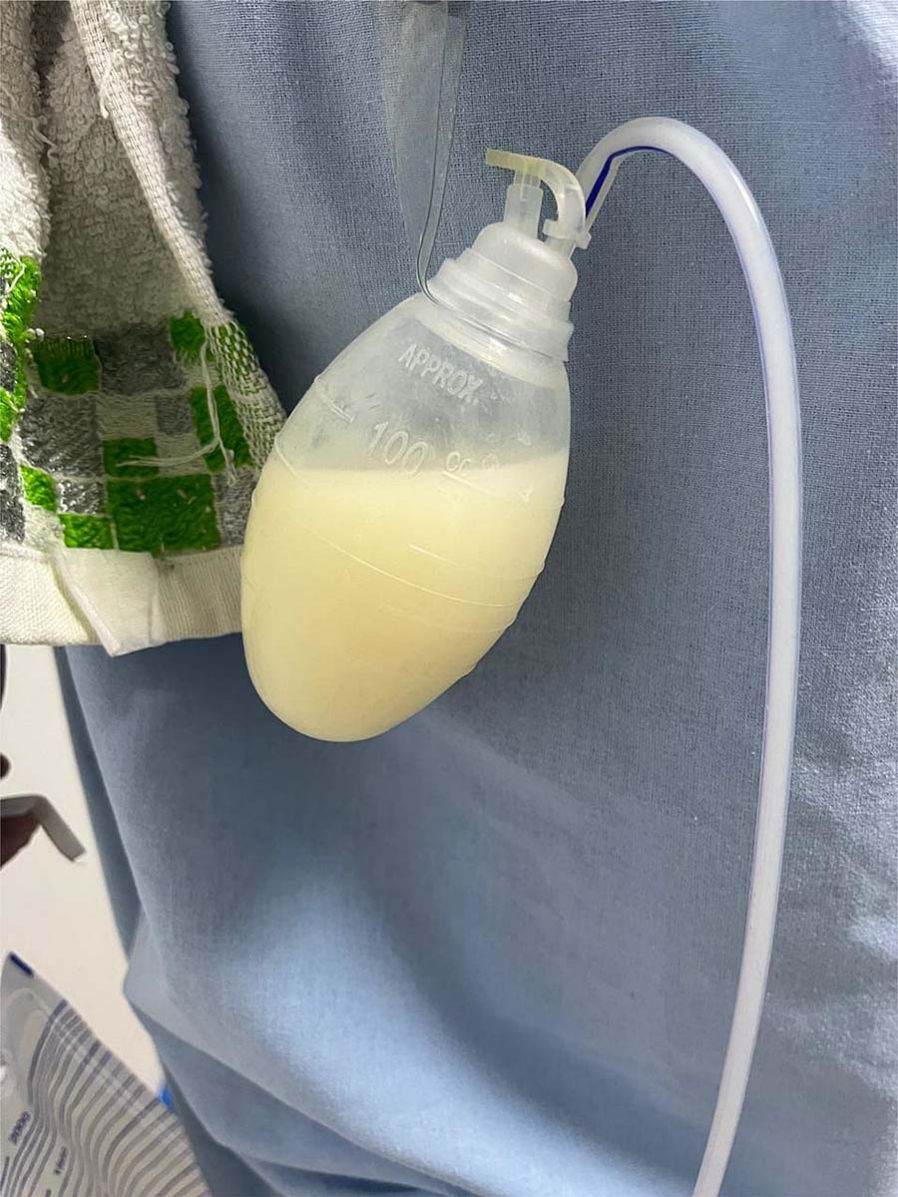
Drainage with milky characteristics.

**Figure 2:**
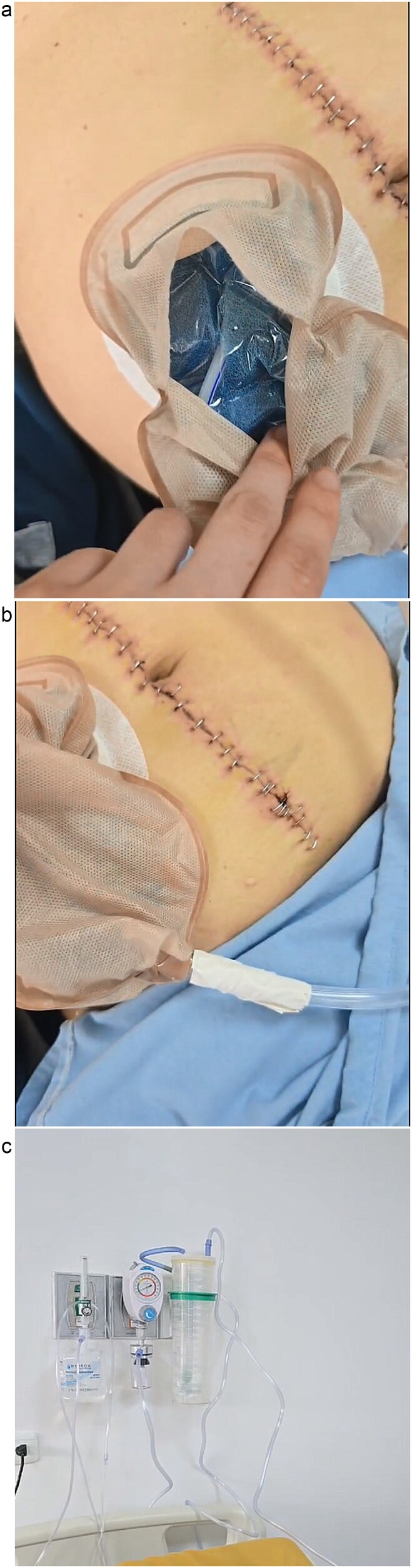
(a) Suction sponges in ostomy bag. (b) Suction hose connection to ostomy sheath. (c) Suction system connection to wall with negative pressure.

On the fifth postoperative day, the patient presented increased drainage production, reaching up to 1300 cc in 1 day, with changes in characteristics from serohematic to milky. On the sixth postoperative day (Fig. 1), cytochemical fluid with triglycerides of 300 mg/dl. The patient was managed as a chylous fistula with a diet containing medium-chain fatty acids and the use of a homemade negative pressure system through a Jackson Pratt drain with the help of an ostomy bag inside with sterile macroporous sponges and connection to a wall suction at a pressure of −60 mmHg (Fig. 2a–c). Five days after starting negative pressure therapy and dietary management, complete closure of the chylous fistula is evident.

## Case 2

A 71-year-old male with no medical history and a history of right inguinal hernioplasty was admitted with mild acute pancreatitis of biliary origin. Once the symptoms were resolved, a laparoscopic cholecystectomy was performed with placement of a Jackson Pratt drain, revealing a scleroatrophic gallbladder with thickened walls. The procedure was performed without complications. On the second postoperative day, an increase in drainage output and a change in characteristics from serosanguineous drainage to milky was noted. Therefore, a cytochemical test was requested, which showed triglycerides of 1045 mg/dl and elevated amylase. A diagnosis of chylous fistula with grade B pancreatic fistula was indicated. Conservative management was decided with medium-chain fatty acids and somatostatin analogue (octreotide). The patient was subsequently discharged with low-output drainage and somatostatin analogue on an outpatient basis, with resolution of the fistula on the 12th day after the start of treatment.

## Discussion

Chylous fistula, an infrequent postoperative complication (0.3%–6%) primarily associated with surgeries involving extensive lymph node dissection, represents a significant clinical challenge [3–5]. Its development is directly related to the extent of dissection and the scope of the surgical intervention [5]. Diagnosis is suspected upon a change in drainage to a milky-appearing fluid and is confirmed by measuring triglyceride levels in the fluid, which are typically elevated above 110 mg/dl [6]. Clinically, it presents with abdominal distension and pain, lower limb edema, and a sensation of fullness [4]. Its presence not only complicates the postoperative course but also leads to a deterioration of nutritional and immune status, prolongs hospital stay, and increases morbidity and mortality [6].

The current therapeutic approach is initially based on conservative management, whose three fundamental objectives are to support and improve the patient's nutrition, to limit chyle formation, and to improve the concomitant disease [7]. This management includes a medium-chain triglyceride (MCT) or low-fat diet, as MCTs are absorbed directly into the intestine and transported to the liver, reducing chyle flow [7, 8]. It can be supplemented with total parenteral nutrition (TPN), reserved for cases of refractoriness or calorie-protein deficiency, as it allows for bowel rest and decreases lymph production [7, 9, 10].

Pharmacological therapy with somatostatin analogs, such as octreotide, is another pillar, as it decreases thoracic duct flow and its triglyceride content, indirectly suppressing pancreatic exocrine function [7, 9]. Negative pressure therapy (50–125 mmHg) is also used, as it extracts interstitial exudate, reduces edema, and decreases lymphatic flow [11]. Most patients respond to this dietary management, even without TPN support [5, 12, 13].

To standardize severity, the International Study Group on Pancreatic Surgery defines three grades: Grade A is managed with dietary restrictions; Grade B requires interventions such as enteral or parenteral nutrition, somatostatin analogs, or percutaneous drains, prolonging hospitalization; and Grade C necessitates invasive treatments, ICU admission, and carries a higher mortality risk [7]. The exact timing for considering conservative treatment failure is not defined, but a persistently high output (>1000 ml/day) for >2 to 3 weeks is estimated as a criterion for considering invasive measures [9, 10]. Other criteria for surgical intervention include drainage >2000 ml/day, severe malnutrition, hydroelectrolyte disorders, or infection [14]. Among the invasive options are paracentesis, percutaneous drainage, conventional lymphangiography which, despite being a diagnostic method, is considered to have therapeutic potential as it not only allows identification of the leak site but also enables its closure, with reported success rates of up to 70%, and a more advanced technique, endolymphatic balloon-occluded retrograde abdominal lymphangiography embolization, with embolization using coils, adhesives, and/or sclerosing agents; within surgical interventions, peritoneovenous shunting is proposed [7, 15]. However, there is no established surgical treatment for this entity [6, 7]. Chylous fistula is clinically significant due to its negative impact on postoperative recovery and nutritional status. Conservative management plays a pivotal role as first-line therapy, allowing effective control of chyle leakage while avoiding invasive procedures. Early implementation of dietary and pharmacological measures can significantly reduce morbidity and hospital stay.

## Conclusions

Chylous fistula is an uncommon complication of abdominal surgery in benign disease, with significant consequences for nutritional and immune function. Conservative management remains the cornerstone of treatment, combining dietary modification, pharmacologic therapy, and, more recently, negative pressure therapy. Based on current evidence and clinical experience, surgical intervention should be reserved for cases in which conservative measures fail.
